# Unraveling the critical role of SUMOylation in the governing of tumor immunity

**DOI:** 10.3389/fimmu.2025.1654167

**Published:** 2025-09-29

**Authors:** Xiangfei Liu, Wei Ding, Lu Jiang, Qianming Chen, Xiaobo Luo

**Affiliations:** State Key Laboratory of Oral Diseases, National Clinical Research Center for Oral Diseases, Chinese Academy of Medical Sciences Research Unit of Oral Carcinogenesis and Management, West China Hospital of Stomatology, Sichuan University, Chengdu, Sichuan, China

**Keywords:** SUMOylation, anti-tumor immunity, MHC-I antigen presentation, JAK/STAT pathway, TAK-981

## Abstract

SUMOylation, a dynamic regulatory process in post-translational modifications (PTMs) mediated by small ubiquitin-like modifier (SUMO) ligases and deSUMOylases, regulates protein function through reversible lysine conjugation. Emerging evidence has identified tumor-mediated hijacking of SUMOylation in both malignant cells and immune components as a novel immune evasion mechanism. This review represents a comprehensive update on how tumor-intrinsic SUMOylation modulates tumor immunity-related JAK/STAT, MHC-I, NF-κB, IFN-I/II pathways and other key proteins to drive its immune evasion, and immune cell-intrinsic SUMOylation in regulating natural killer (NK) and T cell cytotoxicity, dendritic cell (DC) maturation, and macrophage polarization. Tumor immunotherapy is a new potential strategy for cancer, mainly represented by immune checkpoint inhibitions (ICIs), which exhibits poor efficacy in head and neck squamous cell carcinoma (HNSCC), pancreatic ductal adenocarcinoma (PDAC) and other solid tumors. Targeting SUMOylation of tumors presents high potential to synergistically improve the therapeutic effect of ICIs. Preclinical studies have shed light on the therapeutic potential of the combination of SUMOylation inhibitors such as TAK-981 or 2-D08 with ICIs, thus significantly improving tumor prognosis. As current phase I trials suggest dose-dependent toxicity of TAK-981, there is a need for targeted delivery systems; AI-assisted screening of novel SUMOylation inhibitors (SUMOi) which are FDA approved serves as another potential approach; besides, antibodies against these pivotal SUMOylated molecules in tumors could be conjugated with SUMOi to restore the activity of specific proteins in tumor microenvironment. In all, our review proposes that current or other novel strategies for SUMOylation inhibition stands as a promising adjuvant to immunotherapy for tumor management, thereby potentially contributing to the favorable prognosis of cancer patients.

## Introduction

Recently, numerous studies have indicated that post-translational modifications (PTMs) of proteins, including methylation, SUMOylation, phosphorylation, acetylation, lactylation, and glycosylation, harbors high potential to regulate various aspects of cancer progression. These modifications significantly influence tumor growth, metastasis, dysregulated metabolism, and evasion from immune surveillance (1–4). SUMOylation is a dynamic and reversible ubiquitin-like modification that preserves the structural integrity and functional homeostasis of proteins, and the process is accomplished by covalent conjugation of small ubiquitin-like modifier (SUMO) to lysine residues (Lys/K) on substrate proteins (5). Interestingly, growing evidence has shown that hyperactivation of SUMOylation is a hallmark of cancer, with elevated protein SUMOylation levels observed in most cancers (6–8). In particular, SUMOylation of nuclear proteins, the major target of SUMO, serves as a master regulatory hub governing most nuclear processes and diverse cellular programs, including DNA damage repair, transcription regulation, apoptotic machinery, cytokine secretion, and modulation of oncogenes and tumor suppressor genes, thereby modulating tumor growth, migration, inflammation, and angiogenesis signals to mediate its progression (7, 9–11). However, it seems likely that the influence of SUMOylation on tumor suppression or progression is cancer context-dependent. For instance, sentrin-specific protease 1 (SENP1) promotes hepatocarcinogenesis and enhances the stemness of hepatoma cells by reinforcing the deSUMOylation of hypoxia-inducible factor-1 (HIF-1α), thus increasing the stability and transcription of HIF-1α (12). DeSUMOylation of signal transducer and activator of transcription 3 (STAT3) induced by SENP3 improves its transcriptional and oncogenic potential in head and neck cancers (13). In contrast, deSUMOylation of β-catenin may inhibit the expansion of myeloma by accelerating the degradation of β-catenin via the ubiquitin-proteasomal system, thereby downregulating the Wnt/β-catenin pathway (14). In addition, SUMO modification of the NOP2/Sun domain (NSUN2) facilitates its carcinogenic activity by stabilizing NSUN2 and enabling its nuclear trafficking (15). Similarly, SUMOylation of Mouse double minute 2 homolog (MDM2) accounts for the development of colon or prostate cancer by triggering p53 degradation (16, 17).

The immune system serves a pivotal function in safeguarding human physiological integrity when undergoing attack by foreign invasion, including the early recognition of tumor antigens, which is supported by the clinical observation that immunodeficient patients have a higher risk of cancer (18). Hence, the intactness of anti-tumor immunity is critical for the elimination of tumors. Anti-tumor immunity is generally comprised of innate immunity, which is the non-specific defense against tumor predominantly directed by neutrophils, macrophages and natural killer cells (NKs), and adaptive immunity, which is specific and exerts more robust killing of cancer cells, such as cellular immunity primarily executed by cytotoxic T cells (19, 20). Given this, tumor immunotherapy is becoming an emerging and promising approach for the management of tumors and has revolutionized the traditional treatment efficacy, represented by the application of immune checkpoint inhibitions (ICIs); however, limited efficacy has been observed in patients with pancreatic ductal adenocarcinoma (PDAC) and head and neck squamous cell carcinoma (HNSCC) (21, 22). To seek for the potential reason, it might be largely attributed to the evolving mechanisms of tumors to avoid immune killing, such as the impaired MHC-I antigen presentation in HNSCC (23). Notably, studies have indicated that SUMOylation of proteins within cancer cells or immune cells plays an important role in modulating anti-tumor immunity, including the JAK/STAT, MHC-I antigen presentation, IFN-I/II pathway and so forth (24–28). It is of great value to dissect the key mechanisms underpinning SUMOylation-mediated tumor immune escape, thus seeking more potent strategies to augment immunotherapeutic efficacy. In this review, we briefly discuss the routine process of SUMOylation and focus on the molecular regulatory mechanisms by which SUMOylation of certain proteins wrestles anti-tumor immunity.

## Overview of physiological processes of SUMOylation

**Figure 1:**
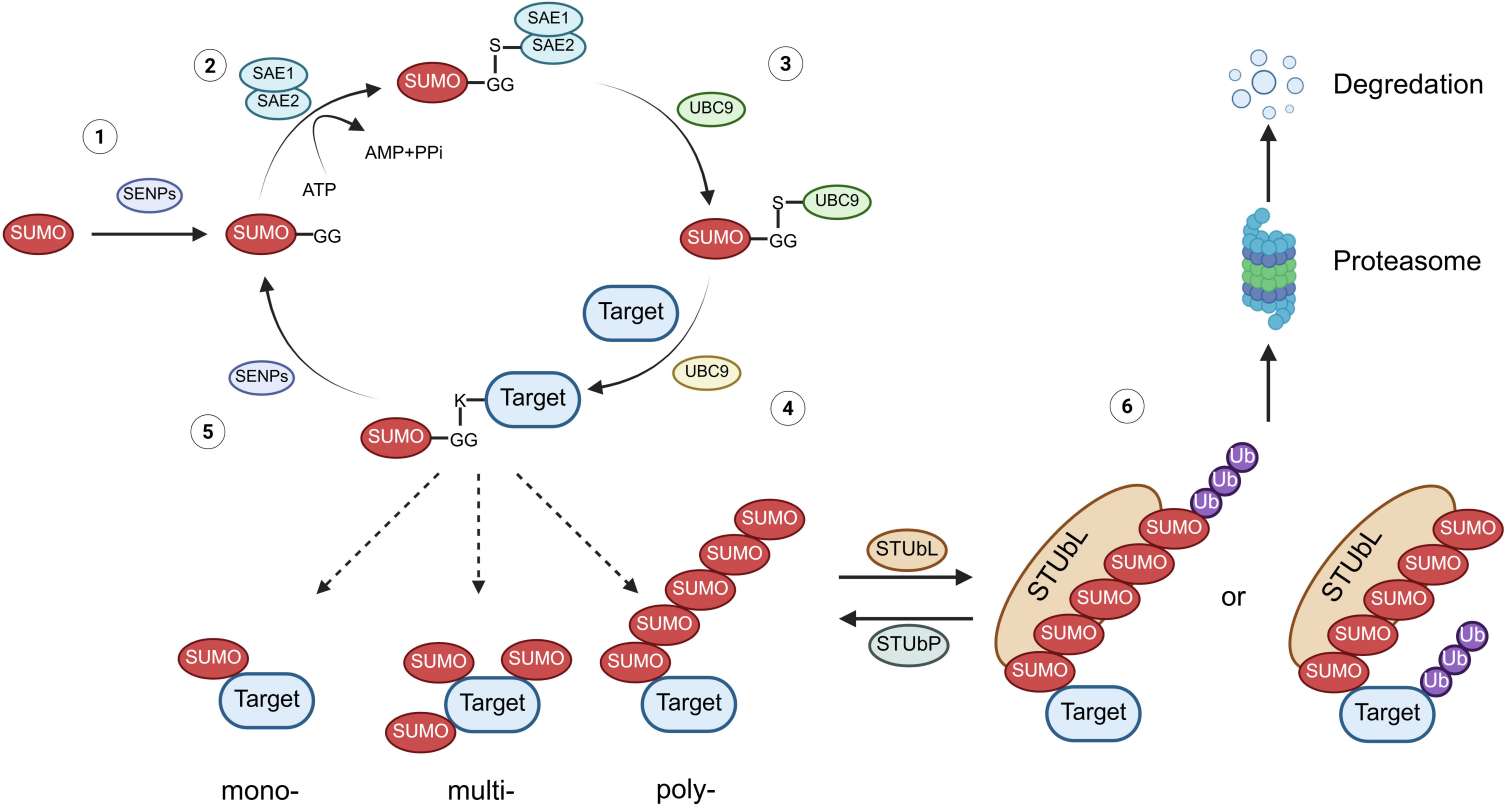
The physiological process of protein SUMOylation. (1) Maturation: The C-terminal Gly-Gly (G-G) motif of the SUMO protein is exposed after cleavage by SENPs. (2) Activation: The mature SUMO protein forms a thioester bond with SAE1/2 (E1) in an ATP-dependent manner. (3) Conjugation: The SUMO protein is transferred from E1 to the SUMO-conjugating enzyme UBC9 (E2). (4) Ligation: SUMO is covalently attached to lysine residues on the substrate protein, resulting in mono-, multiple-, or poly-SUMOylation. (5) DeSUMOylation: At various stages, SUMO can be cleaved from the substrate protein by SENPs or other deSUMOylation enzymes, thus reversing the modification. (6) Ubiquitination: Poly-SUMOylation of substrate proteins is recognized by STUbLs, leading to their subsequent degradation via the proteasomal pathway. Conversely, ubiquitination of these substrates can be reversed by STUbPs. SENPs, sentrin-specific proteases; STUbLs, SUMO-targeted ubiquitin ligases; STUbPs, SUMO-targeted ubiquitin-specific proteases.

The process of SUMOylation is a tri-enzymatic cascade with SUMO proteins being bound to target proteins under the catalysis of SUMO E1 activating enzyme (SAE1/2), followed by the single SUMO E2 conjugating enzyme (UBC9), as well as the substrate-specific SUMO E3 ligase, which affects the stability and biological activity, degradation, and orientation biological function of targeted proteins, and a special E4 enzyme named ZNF451 has been identified to facilitate the assembly of SUMO-chains (7, 11, 29) (**Figure 1**). To date, approximately five paralogs of SUMO proteins (SUMO1-5) can be expressed by mammalian cells, among which SUMO2/3 is the most abundant protein that can form chains by connecting with each other (8, 11, 30). Due to the 97% sequence identity of SUMO2 and SUMO3, they are collectively named as SUMO2/3, while SUMO1 has only 53% sequence resemblance with SUMO2/3 (31). Under the catalysis of these enzymes, SUMO is attached to the lysine (Lys/K) of targeted proteins to induce mono-, multi-, and poly-modification (6, 30, 32). Moreover, poly-SUMOylation of the substrate protein can be identified by SUMO-targeted ubiquitin ligase (STUbL, including RNF4 and RNF111), leading to its degradation by ubiquitination, which could be reversed by ubiquitin protease (STUbP, including USP7, USP11, and ATX3, which targets SUMO molecules), highlighting the intricate interplay between SUMOylation and ubiquitination in protein regulation (30, 33). In addition to the enzymes responsible for SUMOylation, some other enzymes are involved in deSUMOylation, named as SUMO-specific protease (SENP), thus removing the SUMOylation modification of targeted proteins. Specifically, six types of SENPs (SENP1/2/3/5/6/7) and the newly identified DESI1, DESI2, and USPL1 enzymes implicate in this process (7).

## The tumor cell-intrinsic SUMOylation in the modulation of anti-tumor immunity

### JAK/STAT pathway

**Figure 2:**
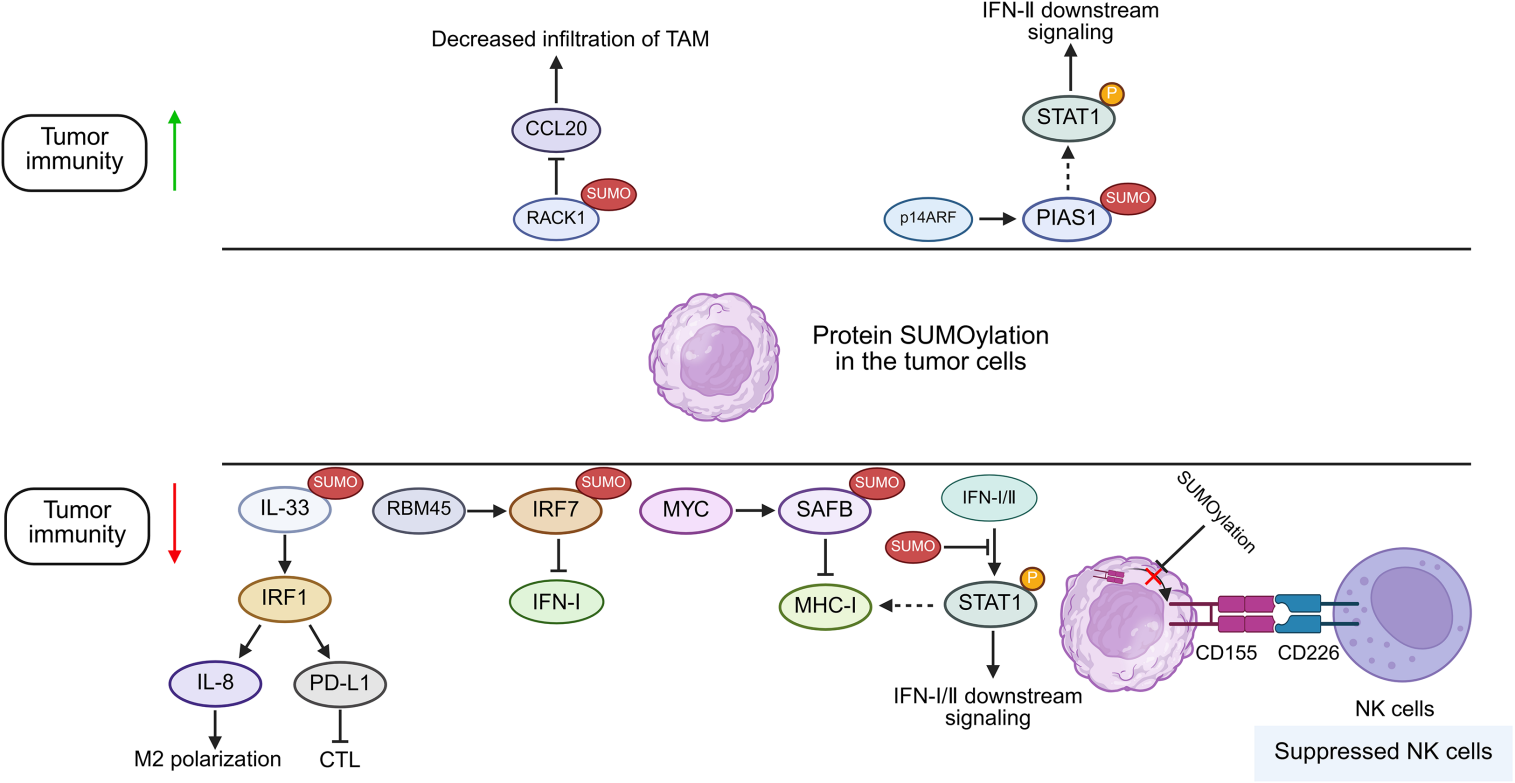
Proposed function of protein SUMOylation in cancer cells in modulating tumor immunity. Increased SUMOylation of targeted proteins in tumor cells can potentiate or suppress anti-tumor immunity by regulating IFN-I/II signaling, the infiltration of CTLs, NK cells and TAMs. RBM45, RNA Binding Motif Protein 45; SAFB, scaffold attachment factor B; MHC-I, major histocompatibility complex I; STAT, signal transducer and activator of transcription; PIAS, protein inhibitor of activated STAT; RACK1, Receptor For Activated C Kinase 1; p14ARF, p14 alternative reading frame; IFN: Interferon; IL-33, Interleukin 33; IRF, Interferon Regulatory Factor; PD-L1, Programmed cell death ligand 1; NK cells, natural killer cells; CTL, cytotoxic T lymphocyte; TAM, tumor associated macrophages.

The protein inhibitor of activated STAT (PIAS) family was initially discovered as a suppressor of STAT in the JAK/STAT pathway by impairing the DNA-binding capacity of its cognate recognition motifs (34, 35). Later, it was revealed that the PIAS family possesses E3 ligase activity, which regulates a variety of proteins via SUMOylation (36). In line with this, some researchers have found that SUMOylation modulates innate immunity by impairing virus-triggered type I interferon (IFN-I) production and disrupting IFN-I/II-dependent STAT1 and STAT2 activation cascades (37, 38). Maarifi et al. reported that SUMO hyperexpression drives covalent SUMO conjugation to STAT1-K703 and results in the impairment of IFN-I/II-triggered STAT1 activation dynamics in several cancers, including human glioblastoma astrocytoma, cervical cancer, and hepatocellular carcinoma, and IFN-I/II plays a negative feedback regulation on its own signal by enhancing the SUMOylation of STAT1 (24) (**Figure 2**). Besides, a recent study revealed that PIAS1 can be SUMOylated by the tumor suppressor p14ARF, which consequently inhibits PIAS1-mediated SUMOylation of STAT1, thereby effectively enhancing the IFN-γ-induced immune response (39). Beyond PIAS1, researchers have identified a protein-coding circular non-coding RNA, circPIAS1, which could be translated into a 108-amino acid peptide (circPIAS1-108aa) (40). This peptide can recruit the SUMO E3 ligase RANBP2 to promote STAT1 SUMOylation, consequently suppressing STAT1 phosphorylation and subsequently facilitating immune escape of melanoma (40). These indicate that during cellular stress responses, SUMOylation-mediated regulation of the JAK/STAT pathway is evolutionarily conserved to prevent excessive activation of the immune system. However, the mechanism that hyperactivated SUMOylation may impair JAK/STAT signaling could be hijacked by cancers to promote its immune evasion.

### MHC-I antigen presentation pathway and MYC

It is generally recognized that sufficient infiltration of T cells, especially cytotoxic T cells, within the tumor microenvironment (TME) is indispensable for the therapeutic efficacy of ICIs for cancers, while the absence of abundant T cells is usually observed in cancers such as HNSCC (23). As MHC-I-associated antigen presentation, including antigen production, activation of T cells, and specific recognition of tumors, is crucial as the first specific signal for T cell activation, the intactness of the pathway is highly appreciated for its normal function (41). Dysfunction of the MHC-I is a well-established cause of both primary and acquired resistance to cancer immunotherapies (42). To date, both JAK/STAT and NF-κB pathways have been shown to be important for upregulating the MHC-I pathway (18). Attenuated MHC-I antigen presentation is frequently observed, among which SUMOylation might partially contribute to its suppression by impairing JAK/STAT pathway based on aforementioned studies. Moreover, the fact that co-administration of TAK981, a specific inhibitor of SUMOylation, and PD-1 blockade markedly enhanced the survival of tumor-bearing mice further supports these findings (43).

*MYC* gene, which is dysregulated in 70% of tumors, serves as a predominant oncogenic driver across human cancers, encoding a family of transcription factors, with the products serving as key regulators of cell proliferation, cell differentiation, cell cycle, and metabolism (44, 45, 86). Thus, targeting of MYC exhibits a wide range of therapeutic effects in tumors. However, MYC is an inherently disordered protein with no stable conformation and no proper site suitable for small-molecule binding. Recently, it has been found that SUMOylation of MYC catalyzed by PIAS1 not only upregulates MYC by preventing its degradation but also promotes MYC phosphorylation, thereby leading to its higher transcriptional activity in B-cell lymphoma (46). Using murine lung and colon cancer models, Kotani’s team demonstrated that pharmacological inhibition of MYC by TAK981 promotes activation of STING-IFN-I pathway with consequent STAT1 phosphorylation and MHC-I upregulation in KRAS-mutant cancer cells (47). Scaffold attachment factor B (SAFB) was previously reported to inhibit MHC-I in an SUMOylation-dependent manner (81). Demel further validated that MYC promotes SUMO2/3 modification of SAFB in several cancers, encompassing B lymphoma and osteosarcoma, consequently inhibiting MHC-I expression and inducing tumor immune escape (8, 48). Moreover, they found that TAK981 could reverse this process via reverting MHC-I expression, thereby augmenting anti-tumor immunity (8). Thus, inhibiting SUMOylation of MYC and its downstream proteins may alternatively inhibit the oncogenic function of MYC and promote adaptive immune responses in cancers.

### NF-κB signaling

Accumulating evidence indicates that NF-κB signaling is not only crucial for the occurrence, proliferation, differentiation, apoptosis, invasion, and metastasis of cancer cells but also modulates tumor immunity. For instance, one study on lung cancer indicated that activation of NF-κB augments regulatory T cell (Treg) ontogeny as well as functional polarization, whose tumor infiltrative capacity correlates with adverse clinical outcomes (49). Similarly, zhou et al. suggested that NF-κB may promote the expression of chemokines in lung cancer, such as cytokine ligand 2 (CCL2), thus facilitating monocyte recruitment and infiltration of immunosuppressive TAMs to the tumor bed (50). In contrast, Matthew et al. found that stimulation of T cell-intrinsic canonical NF-κB enhanced its activation and effector function, further increasing the clonal expansion of tumor-infiltrated CD8+ T cells (51). Moreover, NF-κB contributes to the transcriptional regulation of MHC-I in neoplastic populations (18).

Notably, multiple recent studies have revealed that SUMOylation might regulate this pathway by interacting with several related key proteins. For example, the SUMOylation of certain proteins can stimulate the NF-κB pathway. Nuclear receptor 4A (NR4A1) participates in suppressing NF-κB signaling induced by IL-1β and TNF-α, but SUMOylated NR4R1 precipitates its ubiquitin-proteasomal turnover, thus upregulating the NF-κB pathway in HeLa and Jurkat cells (52, 53). Sophia et al. pointed out that SUMOylation of TNF receptor-related factor 3 (TRAF3) mediated by UBC9 results in its association with the CD40 receptor, which ultimately leads to the degradation of TRAF3 and non-canonical NF-kB activation in HeLa cell (54). In addition, SUMOylated NEMO is essential for its translocation to the cytoplasm and further induction of NF-κB activation (55). In contrast, the RWD-containing SUMO enhancer (RSUME), by interacting with the SUMO E2 ligase UBC9, could promote covalent SUMO modification of IκBα, leading to suppression of NF-κB transcription output (56). However, the functional consequences of SUMOylation-mediated NF-κB signaling on oncogenic progression remain to be comprehensively validated across heterogeneous malignancies using *in vivo* preclinical models. A study by Liu et al. et al. demonstrated that SUMOylation of MANF can promote its nuclear translocation, and SUMOylated p65 then binds to nuclear MAFN, thus inhibiting NF-κB pathway activation and liver cancer development through epithelial-mesenchymal transition (EMT) (57).

### PVR

Poliovirus Receptor (PVR), designated as CD155, has dual functions in anti-tumor immunity; on the one hand, CD155 could induce tumor killing by NK cells via associating with CD226; on the other hand, CD155 suppresses NK cell-mediated tumor immunosurveillance through engagement of T cell immunoreceptors with Ig and ITIM domains (TIGIT) (58, 85). Recently, researchers revealed that SUMOylation of CD155 could inhibit its translocation from the cytosol to the cell membrane of multiple myeloma cells, thus attenuating the anti-tumor effect of NK cells (59). This finding implies that SUMOylation inhibitors (SUMOi) could be combined with ICI-based immunotherapies to produce more potent anti-tumor effects.

### IRF7

Interferon regulatory factors (IRFs) are a family of transcription factors that play key roles in regulating interferon expression and governing host immune responses, cell differentiation, and immune regulation (78). In the antiviral response, SUMOylation of IRF3 and IRF7 reduces their transcriptional activity, consequently inhibiting IFN-I production (38). Recent study has further found that in breast cancer, RNA-binding protein 45 (RBM45) knockout reduces IRF7 SUMOylation, which relieve its inhibitory effect on IRF7 and promote IFN-I production (60).

### RACK1

As an evolutionarily conserved scaffolding protein, receptor for activated C kinase 1 (RACK1) plays dual roles in regulating tumor progression and simultaneously remodeling the TME (75). Wang and colleagues revealed that RACK1 can be deSUMOylated by SENP3 for its stabilization, which drives CCL20 expression by potentiating the RACK1/eIF4E axis, consequently promoting infiltration of tumor-associated macrophage (TAM) while suppressing recruitment of cytotoxic T-cells, and ultimately facilitating immune evasion of hepatocellular carcinoma (61).

### IL-33

Interleukin-33 (IL-33) is a member of the IL-1 family, which can function as a nuclear factor within the nucleus while also being released extracellularly to act as a cytokine (80). In hepatocellular carcinoma (HCC), SUMOylation of IL-33 prevents the ubiquitination and degradation of IRF1, which promotes the expression of IL-8 and PD-L1, thus suppressing the anti-tumor activity of macrophages and T cells (62). These explain how SUMOylation contributes to an immunosuppressive phenotype within the TME.

## The function of immune cell-derived SUMOylation in modulating anti-tumor immunity

**Figure 3:**
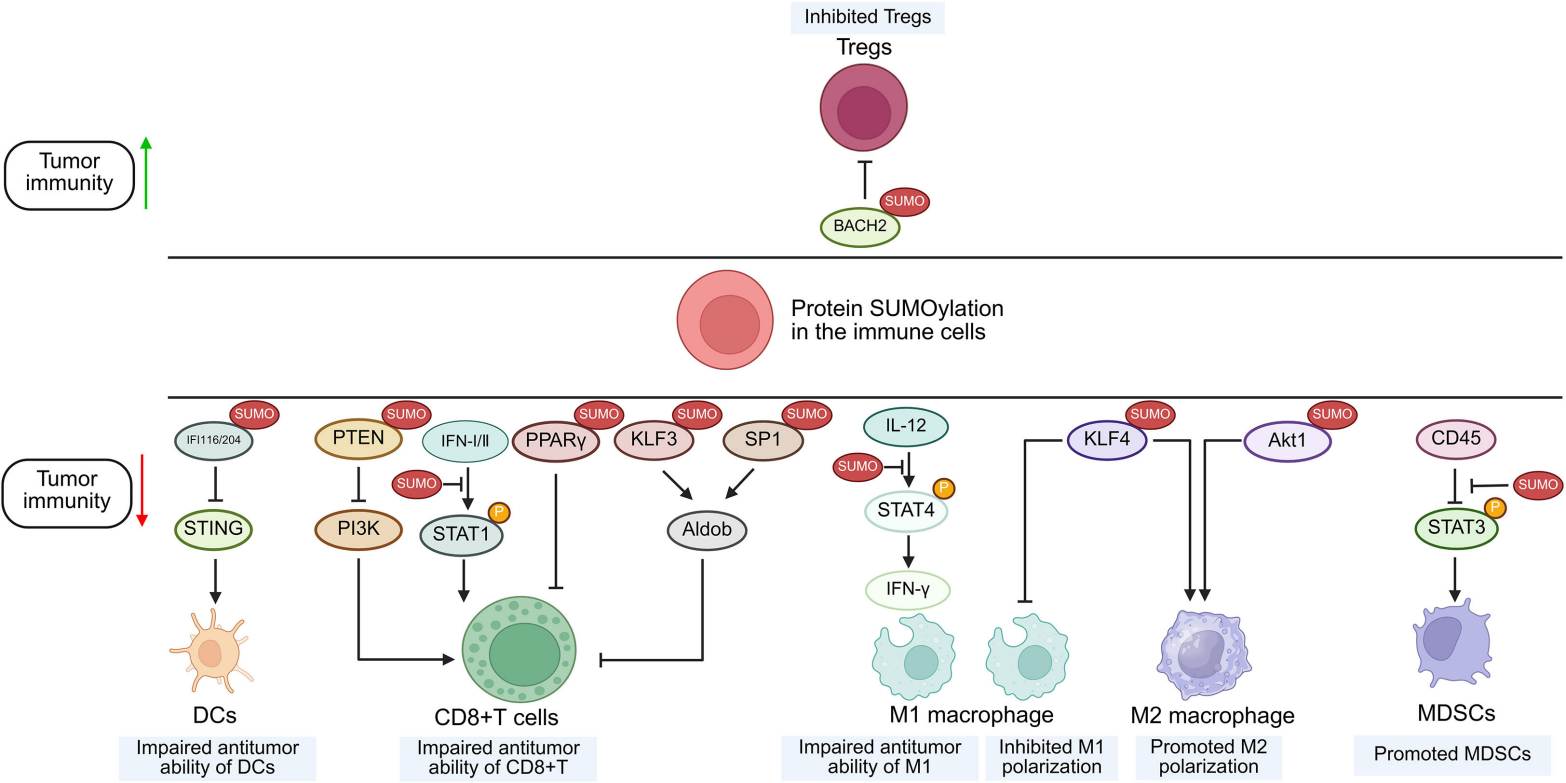
Suggested role of protein SUMOylation within immune cells in the regulation of tumor immunity. Increased SUMOylation of targeted proteins in immune cells can regulate anti-tumor immunity through modulating the function of immune subsets including CD8+ T cells, MDSCs, DCs, M1/M2 macrophages and Tregs. SUMO, small ubiquitin-like modifier; STAT, signal transducer and activator of transcription; KLF4, Krüppel-like factor 4; IFI, interferon-inducible protein; STING, stimulator of interferon genes; BACH2, BTB domain and CNC homolog 2; DCs, dendritic cells; M1, type 1 macrophage; M2, type 2 macrophage; MDSCs, myeloid-derived suppressor cells; Tregs, regulatory T cells.

While tumor cell-intrinsic SUMOylation acts as a pivotal role in regulating anti-tumor immune responses, emerging evidence suggests that SUMOylation of key proteins within immune cells also significantly modulates their anti-tumor activity (**Figure 3**).

### JAK/STAT pathway

Beyond regulating the JAK/STAT pathway in malignant cells, SUMOylation also suppresses the signaling in CD8^+^ T cells. This functional inhibition has been substantiated by multiple studies demonstrating that TAK-981 treatment upregulates phosphorylated STAT1 (p-STAT1) and interferon-stimulated genes (ISGs) in CD8^+^ T lymphocytes (8, 63). Furthermore, in macrophages, UBC9 deficiency triggers deSUMOylation of STAT4, which promotes the secretion of IFN-γ by macrophage activation and subsequently enhances T cell-mediated anti-tumor responses, ultimately suppressing prostate tumorigenesis (64).

### Akt1, IFI204/16 and BACH2

As previously mentioned, SENP3, a specific enzyme for deSUMOylation, is implicated in multiple cancer types through its aberrant expression or dysfunction (65). Numerous experimental studies have revealed that ROS induces the accumulation of SENP3 in different immune cells (27, 66, 67, 83, 84), but their influences on tumor immunity are conflicting. Contrary to the SUMOylation-dependent suppression of STAT1 phosphorylation, SENP3 deficiency in macrophages enhances Akt1 SUMOylation to promote its phosphorylation and activation (68). This cascade drives M2 polarization, ultimately facilitating proliferation and migration of breast cancer (68). In dendritic cells, the accumulation of SENP3 deSUMOylates IFI204 and IFI16, thus activating STING-dependent cytosolic DNA sensing and potentiating the STING-dependent anti-tumor activity of dendritic cells (DCs) (84). In contrast, overexpression of SENP3 could stabilize Treg cells by facilitating deSUMOylation of BACH2, thus contributing to tumor immunosuppression and progression of melanoma, implying that part of the anti-tumor effect of ROS scavengers is exerted through the SENP3/BACH2 axis by inhibiting ROS-induced SENP3 accumulation (27).

### KLF4, KLF3 and SP1

Krüppel-like factors (KLFs) constitute a subgroup of evolutionarily conserved zinc-finger containing transcriptional regulators, among which KLF4 is critical for M2 macrophage polarization (79). Wang et al. demonstrated that SUMOylated KLF4 stabilizes IL-4-driven M2 phenotypic commitment, whereas deSUMOylation of KLF4 regulates M1 polarization of macrophages and enhances the anti-tumor activity of macrophages (76). Furthermore, chromobox protein 4 (CBX4) promotes the SUMOylation of KLF3 and transcription factor 1 (SP1), enhancing their stability and thereby promoting aldolase B (Aldob) transcription, which suppresses CD8+ T cell function by inhibiting the PI3K/Akt pathway (69). Besides, in genetic SUMOylation-deficient KLF1 mouse, the numbers of tumor-infiltrating nature killer T cells and CD8+T cells are increased, resulting in enhanced anti-tumor responses (70).

### PTEN

PTEN, a tumor suppressor and primary negative regulator of PI3K, is frequently downregulated, deleted, or mutated across diverse cancers (82). Evidence indicates that under oxidative stress in CD8^+^ T cells, SENP7-mediated deSUMOylation of PTEN promotes its proteasomal degradation, thereby activating the PI3K/mTOR pathway (82). This signaling sustains both oxidative phosphorylation and glycolysis in CD8^+^ T cells, thus curbing growth of colon cancer (71).

### CD45

CD45 is a ubiquitous leukocyte receptor that exhibits tyrosine phosphatase activity. STAT3 serves as a critical regulator of the functional competence of myeloid-derived suppressor cells (MDSC) (72, 73). It has been shown that SENP1 could deSUMOylate CD45 and consequently improve the phosphatase activity of CD45, thus downregulating STAT3 phosphorylation, inhibiting MDSCs, and preventing the progression of melanoma and Lewis lung cancer (26).

### PPARγ

Although PPARγ (proliferator-activated receptor γ) is a major regulator of adipocyte differentiation and function, it also governs immune cell differentiation and function (77). A study indicates that autocrine VEGF-B secretion by T cells promotes SENP2 expression, which inhibits PPARγ SUMOylation, thereby enhancing PPARγ activity and maintaining lipid synthesis, mitochondrial fitness, and T cell activation (74) (**Table 1**).

**Table 1:** Summary regarding the role of SUMOylation of targeted proteins in tumor and immune cells.

Protein	Year of publication	Cell type	SUMO site	SUMO molecules	Function of SUMOylation	Refs.
RACK1	2025	Hepatocellular carcinoma cell	K264, 271	SUMO3	Impairing CCL20 expression and infiltration of TAM	(75)
KLF3 and SP1	2024	CD8+T cell	Unclear	SUMO1	Inhibiting the PI3K/Akt pathway and suppressing CD8+ T cell function	(76)
PPARγ	2024	CD8+T cell	Unclear	SUMO1	Inhibiting PPARγ activity and CD8^+^ T cells	(77)
IRF7	2024	Breast cancer cell	K444, 446,452	SUMO2	Impairing interferon production	(78)
KLF4	2023	M1 and M2	K278	SUMO1	Promoting M1 polarization and inhibiting M2 polarization	(79)
IL-33	2023	Hepatocellular carcinoma cell	K54	SUMO1	Preventing the ubiquitination and degradation of IRF1 and the anti-tumor activity of macrophages and T cells	(80)
SAFB	2022	B lymphoma/osteosarcoma/colorectal carcinoma/breast cancer	Unclear	SUMO1-3	Suppressing the MHC-I pathway	(81)
PTEN	2022	CD8+T cell	Unclear	SUMO2/3	Impairing PI3K/mTOR pathway and CD8^+^ T cells	(82)
IFI16 and IFI204	2021	DCs	IFI204-K83	SUMO2/3	Activating STING-dependent antitumor activity of DCs	(83)
Akt1	2021	Breast cancer cell	Unclear	SUMO2/3	Promoting its phosphorylation and activation and driving M2 polarization	(84)
CD45	2019	MDSC	K867, K77	SUMO1	Promoting the phosphorylation of STAT3 and development and function of MDSCs	(26)
PIAS1	2018	Cancer cells	Unclear	SUMO2/3	Enhancing IFN-γ signaling	(38)
BACH2	2018	Treg cell	K275, 579	SUMO3	Stabilizing Treg cells	(27)
PVR/CD155	2017	Myeloma cells	Unclear	SUMO1	Suppressing the recognition ability of NK cells	(85)
MYC	2016	Lymphoma cell	K51, 52	SUMO1	Promoting MYC phosphorylation and transcriptional activity	(86)
STAT1	2015	Cancer cells	K703	SUMO1, SUMO3	Impairing IFN-γ signaling	(24)

SUMO, small ubiquitin-like modifier; SAFB: Scaffold attachment factor B; MHC-I, major histocompatibility complex I; PIAS, protein inhibitor of activated STAT; IFN-γ, Interferon-γ; PVR, poliovirus receptor; STAT, signal transducer and activator of transcription; KLF, Krüppel-like factor; IFI, interferon-inducible protein; BACH2, BTB Domain And CNC Homolog 2; NK cells, natural killer cells; M1: Type 1 macrophages; M2: Type 2 macrophages; MDSCs, myeloid-derived suppressor cells; RACK1, Receptor For Activated C Kinase 1; PPARγ, Peroxisome Proliferator Activated Receptor Gamma; SP1, Specificity Protein 1; IRF, Interferon Regulatory Factor; IL-33, Interleukin 33; PTEN, Phosphatase And Tensin Homolog.

## The reinforcement of anti-tumor immunity by SUMOylation inhibition

As SUMOylation functions as a master regulatory hub governing the anti-tumor immune response, it is of significant interest to target SUMOylation for tumor interventions. As mentioned previously, SUMOylation of STAT1 in both tumor cells and immune cells can inhibit IFN-I/II-induced phosphorylation of STAT1, thereby promoting immune escape in various tumors (8, 24). Besides, SUMOylation of PTEN inhibits its ubiquitination and subsequent degradation, leading to impaired anti-tumor function of CD8+ T cells and consequently promoting the development of colon cancer (71). Therefore, based on SUMOylation inhibition, targeting the crosstalk between SUMOylation and phosphorylation or ubiquitination of these molecules can enhance anti-tumor immune responses. For instance, Kumar et al. found that SUMOi harbors the potential to increase phosphorylation of STAT1, thereby enhancing the activation of the IFN-I pathway and boosting the anti-tumor immune response (63). Thus, several inhibitors of SUMO E1 and E2 enzymes, naturally derived or artificially produced, have been identified or developed. Since natural E1 inhibitors can also induce some unintentional biological effects and primarily function in the micromolar range, these are less applied in clinical trials, however, synthetic SUMOi exhibits more specific effects and potent efficacy (87). ML-792 and TAK-981 belong to synthetic E1 inhibitors, which inhibit E1 activity by forming an irreversible adduct with SUMO (87, 88). And a novel, orally bioavailable E1 enzyme inhibitor named as SB-4826 has been developed, which forms an irreversible bond with the E1 enzyme (89, 90). In contrast, E2 inhibitors function by either binding to the E2 enzyme to suppress the formation of the E2-SUMO conjugate, as demonstrated by spectomycin B1, or by preventing the transfer of SUMO from UBC9-SUMO to substrate proteins, as observed with 2-D08 (87, 88). Presently, several synthetic SUMOylation inhibitors are undergoing preclinical or clinical evaluations for their potential in boosting the anti-tumor immune responses.

The 2-D08, as an inhibitor of the SUMO E2 enzyme, could enhance the anti-tumor potential of tumor-associated macrophages (TAMs) upon intratumoral injection in a prostate cancer mouse model; as PD-1 expression on CD8+ T cells was simultaneously upregulated, combining 2-D08 with ICI significantly suppressed the tumor growth (64).

SB-4826 treatment suppresses tumor growth in various tumor models, such as A20 lymphoma and CT-26 colorectal cancer models, and its efficacy is markedly enhanced when combined with ICIs therapy (89, 90). However, due to the limited research and insufficient evidences, only TAK-981 has been utilized in clinical trials.

TAK-981, as a specific suppressor of SUMO E1 enzyme, has been revealed to induce broad systemic immunomodulatory effects when administered for cancer therapy. First, TAK-981 can stimulate the innate immune response in murine tumor models. For PDAC with intrinsic immunosuppressive environment, Kumar et al. reported that TAK-981 induced tumor regression through potentiation of IFN signaling within tumor-infiltrating lymphocytes (TILs) and NK cells (63). Similarly, using mouse models of colon cancer and lymphoma, Lightcap et al. demonstrated that TAK-981 activates the IFN-I pathway in an IFNAR1-dependent manner, thereby promoting DCs and T cells’ activation (43). Furthermore, TAK-981 suppresses the tumor-promoting functions of cancer-associated fibroblasts (CAFs) and drives macrophage polarization toward the M1 phenotype, thus inhibiting tumor progression (91). Beyond TAK-981, another preclinical study demonstrates that ML792 similarly remodels the TME in HCC and enhances antitumor immunity (92).

Moreover, the combination of TAK-981 with other treatment modalities have yielded more robust effects in preclinical tumor models. First, TAK-981 in combination with anti-PD1 or anti-CTLA4 therapy could further foster anti-tumor immune response and prolong the survival time of mice (43, 63). Besides, co-administration of TAK-981 with the anti-CD38 antibody daratumumab or anti-CD20 antibody rituximab have potentiated antitumor activity by enhancing macrophage phagocytosis and NK cell cytotoxicity via IFN-I pathway activation in multiple myeloma, diffuse large B-cell lymphoma, and Burkitt lymphoma tumor models (93). In addition to the combination of ICIs and TAK-981, Lu et al. revealed that the application of TAK-981 can resume the levels of CH25H by suppression ATF3 in effector cells, thus augmenting CAR-T therapy and the anti-tumor immunity (94); besides, researchers have integrated TCR therapy with the DNA methylation inhibitor 5-Aza-2’-deoxycytidine and TAK-981, and this triple combination therapy has induced more sustained anti-tumor activity in mouse models of acute myeloid leukemia and multiple myeloma (95).

Except for animal experiments, TAK-981 has entered phase 1 of clinical evaluation (#NCT04381650; www.clinicaltrials.gov). Moreover, four phase1/2 clinical trials (#NCT05976334, #NCT03648732, #NCT04074330, and #NCT04776018) were terminated, and another phase 0 clinical trial (#NCT04065555) was completed (**Table 2**). In a clinical trial of #NCT04065555, 12 patients with HNSCC were administered with TAK-981 in a microdose through percutaneous intratumor injection to directly evaluate the effect of TAK-981 on the tumors. The authors revealed that TAK981 induces immune-favorable remodeling of the TME primarily through orchestrated activation of IFN-I/II signaling, which influences multiple components of the TME in a dose-dependent manner, including cancer-associated fibroblasts, immune cells, and cancer cells (96). Furthermore, they found that TAK-981 induced M1 polarization of macrophages and recruitment of cytotoxic T cells (96). These results are partially consistent with the terminated phase 1/2 clinical trials (#NCT03648732), which demonstrated that TAK-981 can induce activation of the IFN-I transcriptional program and increase the number of activated NK, CD8, and CD4 T cells (97, 98). Notably, systemic use of TAK981 have exhibited certain side effects. In a clinical trial (#NCT03648732), four dose-limiting toxicities were observed: ALT/AST elevation, pneumonitis, stomatitis, and cognitive disturbance (97). Except for these, the predominant treatment-emergent adverse events (TEAEs) with an incidence rate of more than 20% are pyrexia, diarrhea, headache, nausea, fatigue, vomiting, and decreased appetite; common TEAEs of its application ≥grade 3 were hypokalemia, anemia, decreased lymphocyte count, and abdominal pain, with an incidence rate of more than 5% (97). Hence, the results of these clinical trials further confirm the findings of these animal model studies, implying that TAK-981 can not only stimulate the anti-tumor activation of innate lymphoid cells, including DCs, macrophages, and NK cells, but also modulate the adaptive immune response, covering T cell infiltration and function, and inhibiting the functions of Tregs and MDSCs. Considering the inevitable side effects of systemic TAK981, the local administration of low-dose TAK981 may serve as an optimal alternative approach in future trials.

**Table 2:** Clinical trials investigating the therapeutic potential of TAK981 for tumors.

Trials identifier	Tumor type	Nation	Initiation year	No. of participants	Phase	Trial status	Outcomes
NCT05976334	Advanced or Metastatic Solid Tumors	Hungary	2023	3	I	Terminated	–
NCT04776018	Relapsed or Refractory Multiple Myeloma	United States	2021	27	I/II	Terminated	Serious adverse events include febrile neutropenia, cytokine release syndrome, pneumonia, hypercalcemia, acute kidney injury, acute respiratory failure and respiratory failure.
NCT04065555	Head and Neck Cancer	United States	2020	12	0	Completed	–
NCT04381650	Select Advanced or Metastatic Solid Tumors	United States	2020	49	I/II	Active, not recruiting	–
NCT04074330	Relapsed or Refractory CD20-Positive Non-Hodgkin Lymphoma	United States	2019	38	I/II	Terminated	The clinical activity of the combination of TAK-981 and rituximab (ORR 29%). Serious adverse events include atrial flutter, pyrexia, hypercreatinemia, cytokine release syndrome, diarrhea, pain and cytokine release syndrome.
NCT03648372	Advanced Solid Tumors or Cancers in the Immune System	United States	2018	109	I/II	Terminated	Grade 3 ALT/AST elevation (60mg BIW), grade 3 pneumonitis (90mg BIW), grade 3 stomatitis and grade 3 cognitive disturbance (120mg BIW). 42.1%fatigue, 39.5%nausea, 31.6%headache, 28.9%diarrhea, 27.6%pyrexia, 23.7%vomiting, 22.4%decreased appetite. 120mg BIW might be the maximum tolerated dose.

## Summary and future prospects

Most tumors have evolved various strategies to evade immune surveillance by elevating SUMOylation levels to create a TME conducive to their progression. Our review provides a comprehensive update on recent researches regarding the function of protein SUMOylation in adjusting immune-oncological homeostasis, wherein the dichotomous role of SUMOylation within tumor cells or immune cells has been elucidated. SUMOylation can modulate innate or adaptive immune responses by regulating key protein functions in tumor cells or immune cells, thereby regulating JAK/STAT, NF-kB, IFN-I/II signaling and other molecules or pathways; besides, as various SUMOylation or deSUMOylation enzymes present in different types of immune or tumor cells, and owing to the diversity of targeted proteins in various sort of cells, the function of SUMOylation behaves differently in varied cells such as contributing to impairment or enhancement of anti-tumor immunity which is aforementioned. Moreover, inhibition of SUMOylation can alleviate the functional suppression of T cells, NK cells, and macrophages while also suppressing myeloid-derived suppressor cells (MDSCs), thus enhancing antigen presentation, tumor recognition and cytotoxicity of immune cells. This effect may be linked to the increase of interferon production and activation of interferon responses (28). Besides, the presence of excessive SUMOylation in most tumors suggests the therapeutic potential of disrupting SUMO conjugation cascades as a precision strategy against cancers by using SUMOylation inhibitors.

**Figure 4:**
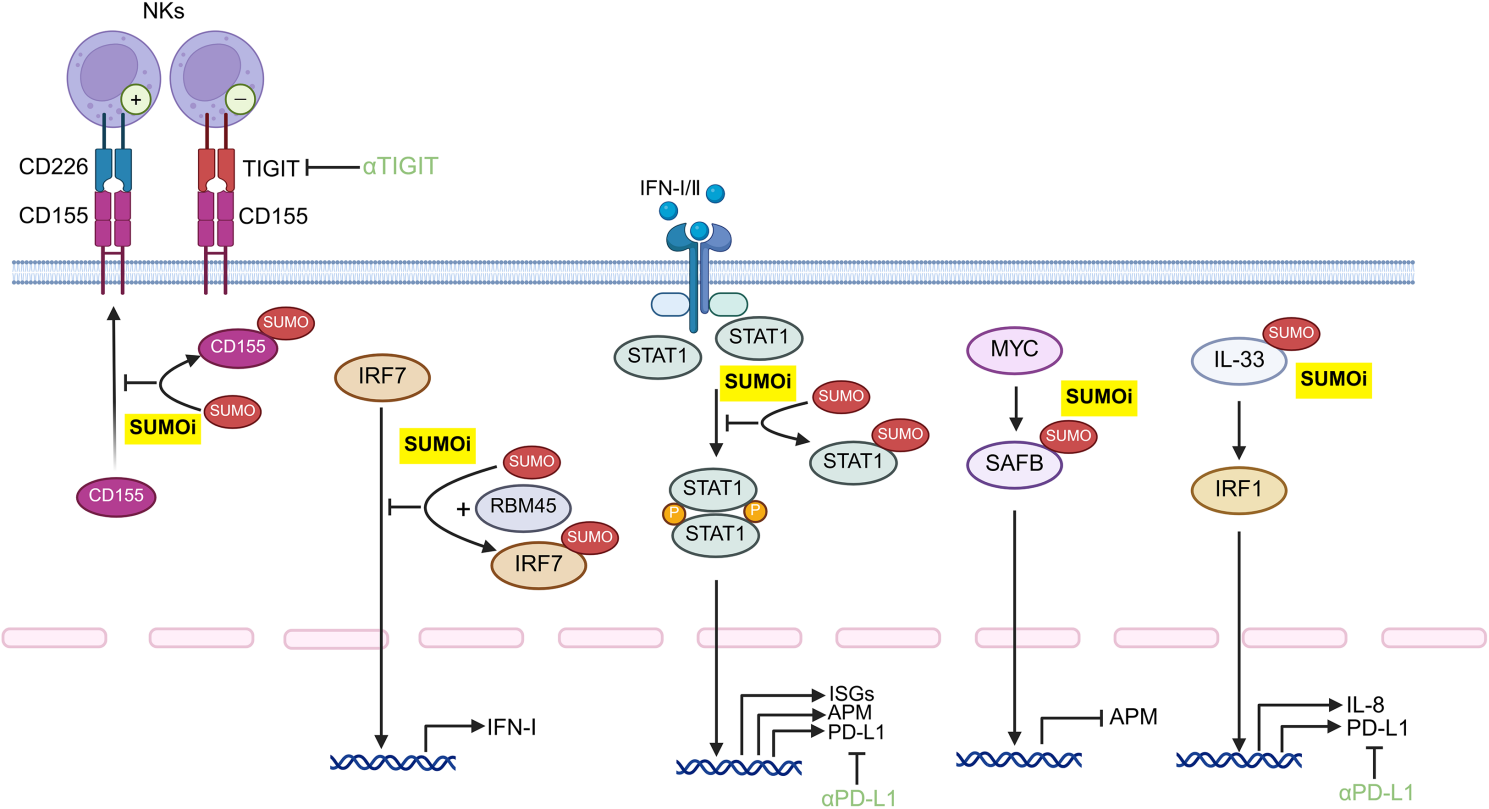
Potential protein target for SUMOylation inhibition within tumor cells for potentiating anti-tumor immune responses. Developing monoclonal antibody-conjugated SUMOi to precisely target distinct tumor-intrinsic proteins may represent a promising strategy to augment anti-tumor immunity, which may orchestrate with the anti-TIGIT or anti-PD-L1 treatment. STAT, signal transducer and activator of transcription; IFN: Interferon; IL-33, Interleukin 33; IRF, Interferon Regulatory Factor; PD-L1, Programmed cell death ligand 1; TIGIT, T cell immunoglobulin and ITIM domain; ISGs, interferon-stimulated genes; APM, antigen-presentation machinery; NK cells, natural killer cells; SUMOi, SUMOylation inhibitors.

**Figure 5:**
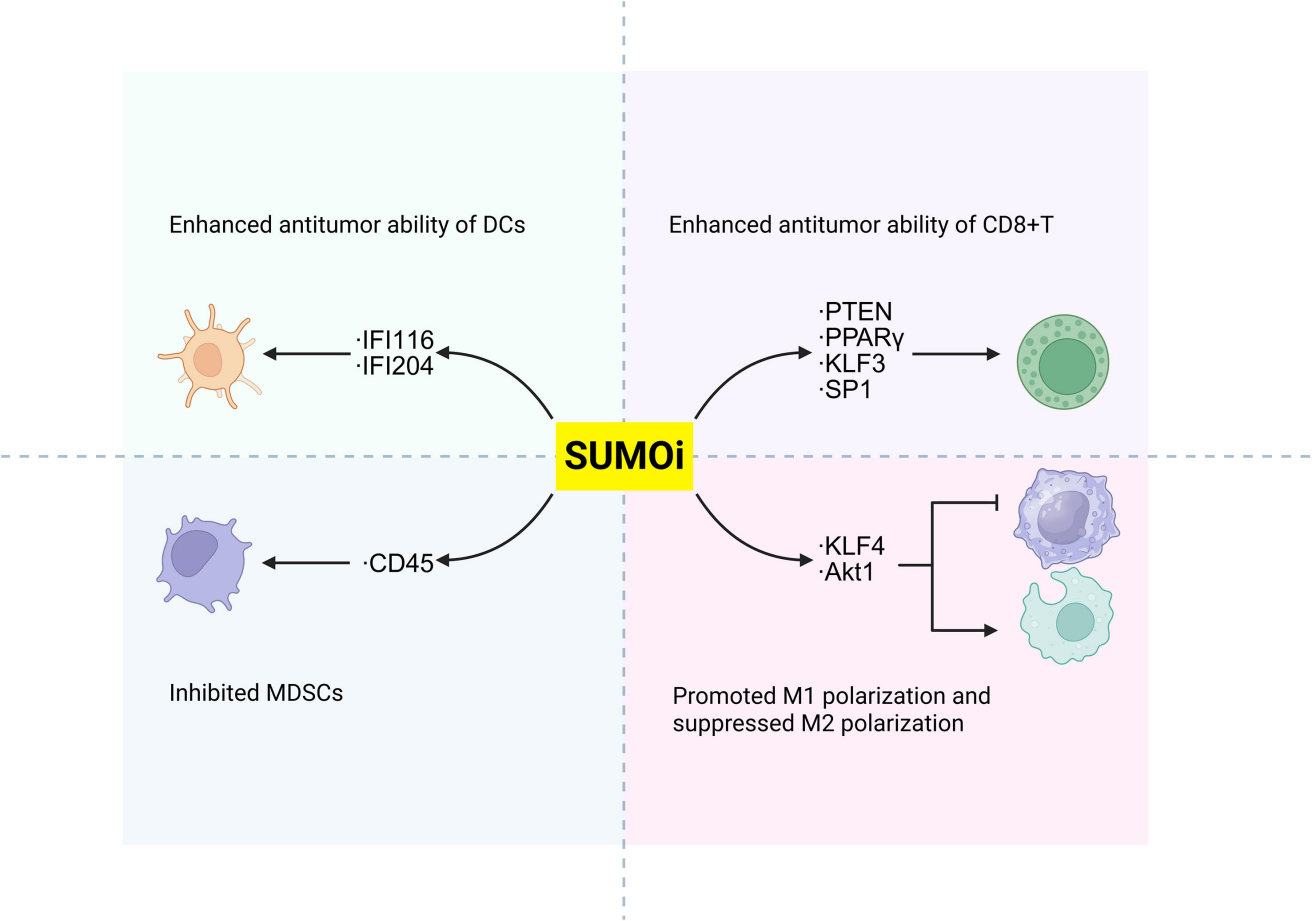
Possible protein target for SUMOylation inhibition in various immune subsets to boost the anti-tumor immunity. The development of monoclonal antibody-conjugated SUMOi for precise target-protein recognition may contribute to enhancing the anti-tumor potential of immune cells. NK cells, natural killer cells; DCs, dendritic cells; M1: Type 1 macrophages; M2: Type 2 macrophages; MDSCs, myeloid-derived suppressor cells; IFI, interferon-inducible protein; PTEN, Phosphatase And Tensin Homolog; PPARγ, Peroxisome Proliferator Activated Receptor Gamma; KLF, Krüppel-like factor; SP1, Specificity Protein 1; SUMOi, SUMOylation inhibitors.

**Figure 6:**
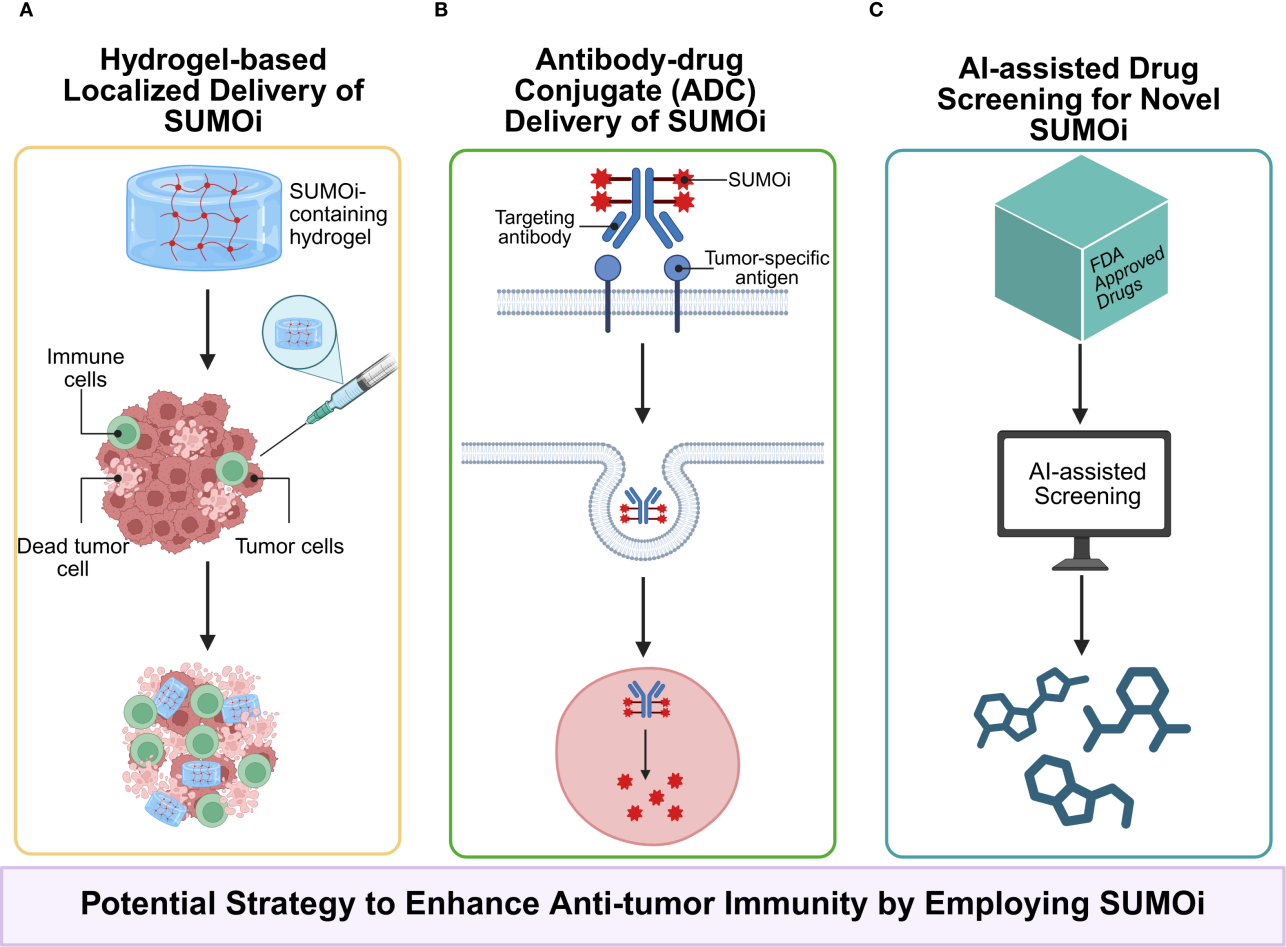
**(A)** Employing local drug delivery systems, such as hydrogels, to carry SUMOi not only minimizes systemic toxicity associated with conventional administration but also enables the sustained release, maintains local drug concentration, and avoids repeated administrations; **(B)** Antibody–drug conjugates (ADCs) enables more precisely targeting of specific proteins in certain cells; **(C)** AI-assisted drug screening from FDA-approved drug library holds promise in identifying novel inhibitors of SUMOylation with reduced toxicity and enhanced compatibility.

The results of those previous preclinical studies indicate that SUMOylation inhibition exhibits partial therapeutic efficacy, possibly owing to the complicated interaction at the tumor-immune interface. Notably, the combination of SUMOi with other immunotherapeutic schemes, such as SUMOi and immune checkpoint immunotherapy, has high potential to facilitate tumor regression, which warrants further clinical studies. However, as SUMOylation plays a versatile role in tumor progression and is cancer-type context-dependent, it is of utmost importance for the development of targeted therapies for inhibiting SUMOylation in certain cells within various tumor types (**Figures 4**, **5**), for example, developing drugs that conjugates mono-antibodies against key SUMOylated proteins in TME with deSUMOylases presents high promise for achieving more precise therapeutic intervention. The combination of SUMOi with anti-PD-L1 for cancer treatment has been extensively studied and well established. We have found that anti-TIGIT therapy may also synergize with SUMOi to enhance anti-tumor immunity. And due to the toxic effects of systemic TAK981, it is necessary to develop a locally delivered strategy for slow-release of TAK981, such as hydrogels with high bio-safety (**Figure 6**). Or utilizing antibody-drug conjugate (ADC) technology to combine tumor cell-targeting antibodies with small molecule SUMOi (such as TAK-981, molecular weight 578.10) may represent a promising strategy to enhance anti-tumor immune responses. In addition, AI-assisted screening of FDA approved therapeutics as potential SUMOi is going to serve as another promising approach. Our review proposes that current or other novel strategies for SUMOylation inhibition present as a promising adjuvant to tumor immunotherapy, upon which better prognosis of patients could be obtained.

## References

[1] Gkountela S Castro-Giner F Szczerba BM Marcus V Julia L Ramona S. Circulating tumor cell clustering shapes DNA methylation to enable metastasis seeding. Cell. (2019) 176:98–112.e14. doi: 10.1016/j.cell.2018.11.046, PMID: 30633912 PMC6363966

[2] Nguyen AT Chia J Ros M Hui KM Saltel F Bard F. Organelle specific O-glycosylation drives MMP14 activation, tumor growth, and metastasis. Cancer Cell. (2017) 32:639–653.e6. doi: 10.1016/j.ccell.2017.10.001, PMID: 29136507

[3] Geffen Y Anand S Akiyama Y Yaron TM Song Y Johnson JL. Pan-cancer analysis of post-translational modifications reveals shared patterns of protein regulation. Cell. (2023) 186:3945–3967.e26. doi: 10.1016/j.cell.2023.07.013, PMID: 37582358 PMC10680287

[4] Boix O Martinez M Vidal S Giménez-Alejandre M Palenzuela L Lorenzo-Sanz L. pTINCR microprotein promotes epithelial differentiation and suppresses tumor growth through CDC42 SUMOylation and activation. Nat Commun. (2022) 13:6840. doi: 10.1038/s41467-022-34529-6, PMID: 36369429 PMC9652315

[5] Rodriguez MS Dargemont C Hay RT. SUMO-1 conjugation *in vivo* requires both a consensus modification motif and nuclear targeting. J Biol Chem. (2001) 276:12654–9. doi: 10.1074/jbc.M009476200, PMID: 11124955

[6] Seeler JS Dejean A. SUMO and the robustness of cancer. Nat Rev Cancer. (2017) 17:184–97. doi: 10.1038/nrc.2016.143, PMID: 28134258

[7] Chang HM Yeh ETH. SUMO: from bench to bedside. Physiol Rev. (2020) 100:1599–619. doi: 10.1152/physrev.00025.2019, PMID: 32666886 PMC7717128

[8] Demel UM Böger M Yousefian S Grunert C Zhang L Hotz PW. Activated SUMOylation restricts MHC class I antigen presentation to confer immune evasion in cancer. J Clin Invest. (2022) 132:e152383. doi: 10.1172/JCI152383, PMID: 35499080 PMC9057585

[9] Zhang T Yang H Zhou Z Bai Y Wang J Wang W. Crosstalk between SUMOylation and ubiquitylation controls DNA end resection by maintaining MRE11 homeostasis on chromatin. Nat Commun. (2022) 13:5133. doi: 10.1038/s41467-022-32920-x, PMID: 36050397 PMC9436968

[10] Eifler K Vertegaal ACO. SUMOylation-mediated regulation of cell cycle progression and cancer. Trends Biochem Sci. (2015) 40:779–93. doi: 10.1016/j.tibs.2015.09.006, PMID: 26601932 PMC4874464

[11] Vertegaal ACO. Signaling mechanisms and cellular functions of SUMO. Nat Rev Mol Cell Biol. (2022) 23:715–31. doi: 10.1038/s41580-022-00500-y, PMID: 35750927

[12] Cui CP Wong CC Kai AK Ho DW Lau EY Tsui YM. SENP1 promotes hypoxia-induced cancer stemness by HIF-1α deSUMOylation and SENP1/HIF-1α positive feedback loop. Gut. (2017) 66:2149–59. doi: 10.1136/gutjnl-2016-313264, PMID: 28258134 PMC5749365

[13] Zhou Z Wang M Li J Xiao M Chin YE Cheng J. SUMOylation and SENP3 regulate STAT3 activation in head and neck cancer. Oncogene. (2016) 35:5826–38. doi: 10.1038/onc.2016.124, PMID: 27181202 PMC5116054

[14] Huang HJ Zhou LL Fu WJ Zhang CY Jiang H Du J. β-catenin SUMOylation is involved in the dysregulated proliferation of myeloma cells. Am J Cancer Res. (2014) 5:309–20., PMID: 25628940 PMC4300696

[15] Hu Y Chen C Tong X Chen S Hu X Pan B. NSUN2 modified by SUMO-2/3 promotes gastric cancer progression and regulates mRNA m5C methylation. Cell Death Dis. (2021) 12:842. doi: 10.1038/s41419-021-04127-3, PMID: 34504059 PMC8429414

[16] Ding B Sun Y Huang J. Overexpression of SKI oncoprotein leads to p53 degradation through regulation of MDM2 protein sumoylation. J Biol Chem. (2012) 287:14621–30. doi: 10.1074/jbc.M111.301523, PMID: 22411991 PMC3340287

[17] Haupt Y Maya R Kazaz A Oren M. Mdm2 promotes the rapid degradation of p53. Nature. (1997) 387:296–9. doi: 10.1038/387296a0, PMID: 9153395

[18] DhatChinamoorthy K Colbert JD Rock KL. Cancer immune evasion through loss of MHC class I antigen presentation. Front Immunol. (2021) 12:636568. doi: 10.3389/fimmu.2021.636568, PMID: 33767702 PMC7986854

[19] Yi M Li T Niu M Mei Q Zhao B Chu Q. Exploiting innate immunity for cancer immunotherapy. Mol Cancer. (2023) 22:187. doi: 10.1186/s12943-023-01885-w, PMID: 38008741 PMC10680233

[20] Xia L Oyang L Lin J Tan S Han Y Wu N. The cancer metabolic reprogramming and immune response. Mol Cancer. (2021) 20(1):28. doi: 10.1186/s12943-021-01316-8, PMID: 33546704 PMC7863491

[21] Ferris RL Blumenschein G Fayette J Guigay J Colevas AD Licitra L. Nivolumab for recurrent squamous-cell carcinoma of the head and neck. N Engl J Med. (2016) 375:1856–67. doi: 10.1056/NEJMoa1602252, PMID: 27718784 PMC5564292

[22] Gautam SK Batra SK Jain M. Molecular and metabolic regulation of immunosuppression in metastatic pancreatic ductal adenocarcinoma. Mol Cancer. (2023) 22:118. doi: 10.1186/s12943-023-01813-y, PMID: 37488598 PMC10367391

[23] Luo X Qiu Y Fitzsimonds ZR Wang Q Chen Q Lei YL. Immune escape of head and neck cancer mediated by the impaired MHC-I antigen presentation pathway. Oncogene. (2024) 43:388–94. doi: 10.1038/s41388-023-02912-2, PMID: 38177410

[24] Maarifi G Maroui MA Dutrieux J Dianoux L Nisole S Chelbi-Alix MK. Small ubiquitin-like modifier alters IFN response. J Immunol. (2015) 195:2312–24. doi: 10.4049/jimmunol.1500035, PMID: 26223657

[25] Sun F Wang FX Zhu H Yue TT Yang CL Luo JH. SUMOylation of PDPK1 Is required to maintain glycolysis-dependent CD4 T-cell homeostasis. Cell Death Dis. (2022) 13:181. doi: 10.1038/s41419-022-04622-1, PMID: 35210408 PMC8873481

[26] Huang X Zuo Y Wang X Wu X Tan H Fan Q. SUMO-specific protease 1 is critical for myeloid-derived suppressor cell development and function. Cancer Res. (2019) 79:3891–902. doi: 10.1158/0008-5472.CAN-18-3497, PMID: 31186231

[27] Yu X Lao Y Teng XL Li S Zhou Y Wang F. SENP3 maintains the stability and function of regulatory T cells via BACH2 deSUMOylation. Nat Commun. (2018) 9:3157. doi: 10.1038/s41467-018-05676-6, PMID: 30089837 PMC6082899

[28] Tharuka MDN Courelli AS Chen Y. Immune regulation by the SUMO family. Nat Rev Immunol. (2025) 25:608–20. doi: 10.1038/s41577-025-01155-4, PMID: 40108400

[29] Eisenhardt N Chaugule VK Koidl S Droescher M Dogan E Rettich J. A new vertebrate SUMO enzyme family reveals insights into SUMO-chain assembly. Nat Struct Mol Biol. (2015) 22:959–67. doi: 10.1038/nsmb.3114, PMID: 26524493

[30] Sriramachandran AM Dohmen RJ. SUMO-targeted ubiquitin ligases. Biochim Biophys Acta. (2014) 1843:75–85. doi: 10.1016/j.bbamcr.2013.08.022, PMID: 24018209

[31] Saitoh H Hinchey J. Functional heterogeneity of small ubiquitin-related protein modifiers SUMO-1 versus SUMO-2/3. J Biol Chem. (2000) 275:6252–8. doi: 10.1074/jbc.275.9.6252, PMID: 10692421

[32] Jansen NS Vertegaal ACO. A chain of events: regulating target proteins by SUMO polymers. Trends Biochem Sci. (2021) 46:113–23. doi: 10.1016/j.tibs.2020.09.002, PMID: 33008689

[33] Liebelt F Vertegaal AC. Ubiquitin-dependent and independent roles of SUMO in proteostasis. Am J Physiol Cell Physiol. (2016) 311:C284–96. doi: 10.1152/ajpcell.00091.2016, PMID: 27335169 PMC5129774

[34] Chung CD Liao J Liu B Rao X Jay P Berta P. Specific inhibition of Stat3 signal transduction by PIAS3. Science. (1997) 278:1803–5. doi: 10.1126/science.278.5344.1803, PMID: 9388184

[35] Liu B Liao J Rao X Kushner SA Chung CD Chang DD. Inhibition of Stat1-mediated gene activation by PIAS1. Proc Natl Acad Sci USA. (1998) 95:10626–31. doi: 10.1073/pnas.95.18.10626, PMID: 9724754 PMC27945

[36] Jackson PK. A new RING for SUMO: wrestling transcriptional responses into nuclear bodies with PIAS family E3 SUMO ligases. Genes Dev. (2001) 15:3053–8. doi: 10.1101/gad.955501, PMID: 11731472

[37] Niu GJ Xu JD Yuan WJ Sun JJ Yang MC He ZH. (PIAS) negatively regulates the JAK/STAT pathway by inhibiting STAT phosphorylation and translocation. Front Immunol. (2018) 9:2392. doi: 10.3389/fimmu.2018.02392, PMID: 30416501 PMC6212522

[38] Hannoun Z Maarifi G Chelbi-Alix MK. The implication of SUMO in intrinsic and innate immunity. Cytokine Growth Factor Rev. (2016) 29:3–16. doi: 10.1016/j.cytogfr.2016.04.003, PMID: 27157810

[39] Alagu J Itahana Y Sim F Chao SH Bi X Itahana K. Tumor Suppressor p14ARF Enhances IFN-γ-Activated Immune Response by Inhibiting PIAS1 via SUMOylation. J Immunol. (2018) 201:451–64. doi: 10.4049/jimmunol.1800327, PMID: 29848755

[40] Zang X He XY Xiao CM Lin Q Wang MY Liu CY. Circular RNA-encoded oncogenic PIAS1 variant blocks immunogenic ferroptosis by modulating the balance between SUMOylation and phosphorylation of STAT1. Mol Cancer. (2024) 23:207. doi: 10.1186/s12943-024-02124-6, PMID: 39334380 PMC11438063

[41] Mellman I Chen DS Powles T Turley SJ. The cancer-immunity cycle: Indication, genotype, and immunotype. Immunity. (2023) 56:2188–205. doi: 10.1016/j.immuni.2023.09.011, PMID: 37820582

[42] Kalbasi A Ribas A. Tumor-intrinsic resistance to immune checkpoint blockade. Nat Rev Immunol. (2020) 20:25–39. doi: 10.1038/s41577-019-0218-4, PMID: 31570880 PMC8499690

[43] Lightcap ES Yu P Grossman S Song K Khattar M Xega K. A small-molecule SUMOylation inhibitor activates antitumor immune responses and potentiates immune therapies in preclinical models. Sci Transl Med. (2021) 13:eaba7791. doi: 10.1126/scitranslmed.aba7791, PMID: 34524860 PMC9719791

[44] Dang CV. MYC on the path to cancer. Cell. (2012) 149:22–35. doi: 10.1016/j.cell.2012.03.003, PMID: 22464321 PMC3345192

[45] Dhanasekaran R Deutzmann A Mahauad-Fernandez WD Hansen AS Gouw AM Felsher DW. The MYC oncogene - the grand orchestrator of cancer growth and immune evasion. Nat Rev Clin Oncol. (2022) 19:23–36. doi: 10.1038/s41571-021-00549-2, PMID: 34508258 PMC9083341

[46] Rabellino A Melegari M Tompkins VS Chen W Van Ness BG Teruya-Feldstein J. PIAS1 promotes lymphomagenesis through MYC upregulation. Cell Rep. (2016) 15:2266–78. doi: 10.1016/j.celrep.2016.05.015, PMID: 27239040 PMC4899214

[47] Kotani H Yamano T Boucher JC Sato S Sakaguchi H Fukuda K. Comprehensive antitumor immune response boosted by dual inhibition of SUMOylation and MEK in MYC-expressing KRAS-mutant cancers. Exp Hematol Oncol. (2024) 13:94. doi: 10.1186/s40164-024-00563-x, PMID: 39334463 PMC11438268

[48] Garee JP Meyer R Oesterreich S. Co-repressor activity of scaffold attachment factor B1 requires sumoylation. Biochem Biophys Res Commun. (2011) 408:516–22. doi: 10.1016/j.bbrc.2011.04.040, PMID: 21527249 PMC3955274

[49] Itahashi K Irie T Yuda J Kumagai S Tanegashima T Lin YT. BATF epigenetically and transcriptionally controls the activation program of regulatory T cells in human tumors. Sci Immunol. (2022) 7:eabk0957. doi: 10.1126/sciimmunol.abk0957, PMID: 36206353

[50] Zhou L Jiang Y Liu X Li L Yang X Dong C. Promotion of tumor-associated macrophages infiltration by elevated neddylation pathway via NF-κB-CCL2 signaling in lung cancer. Oncogene. (2019) 38:5792–804. doi: 10.1038/s41388-019-0840-4, PMID: 31243299

[51] Dong MB Wang G Chow RD Ye L Zhu L Dai X. Systematic immunotherapy target discovery using genome-scale *in vivo* CRISPR screens in CD8 T cells. Cell. (2019) 178:1189–1204.e23. doi: 10.1016/j.cell.2019.07.044, PMID: 31442407 PMC6719679

[52] You B Jiang YY Chen S Yan G Sun J. The orphan nuclear receptor Nur77 suppresses endothelial cell activation through induction of IkappaBalpha expression. Circ Res. (2009) 104:742–9. doi: 10.1161/CIRCRESAHA.108.192286, PMID: 19213954

[53] Zhang L Xie F Zhang J Dijke PT Zhou F. SUMO-triggered ubiquitination of NR4A1 controls macrophage cell death. Cell Death Differ. (2017) 24:1530–9. doi: 10.1038/cdd.2017.29, PMID: 28622293 PMC5563982

[54] Miliara S Gkouskou KK Sharp TV Eliopoulos AG. SUMOylation is required for optimal TRAF3 signaling capacity. PloS One. (2013) 8:e80470. doi: 10.1371/journal.pone.0080470, PMID: 24260396 PMC3832365

[55] Mabb AM Wuerzberger-Davis SM Miyamoto S. PIASy mediates NEMO sumoylation and NF-kappaB activation in response to genotoxic stress. Nat Cell Biol. (2006) 8:986–93. doi: 10.1038/ncb1458, PMID: 16906147

[56] Carbia-Nagashima A Gerez J Perez-Castro C Paez-Pereda M Silberstein S Stalla GK. RSUME, a small RWD-containing protein, enhances SUMO conjugation and stabilizes HIF-1alpha during hypoxia. Cell. (2007) 131:309–23. doi: 10.1016/j.cell.2007.07.044, PMID: 17956732

[57] Liu J Wu Z Han D Wei C Liang Y Jiang T. Mesencephalic astrocyte-derived neurotrophic factor inhibits liver cancer through small ubiquitin-related modifier (SUMO)ylation-related suppression of NF-κB/snail signaling pathway and epithelial-mesenchymal transition. Hepatology. (2020) 71:1262–78. doi: 10.1002/hep.30917, PMID: 31469428 PMC7187412

[58] Lupo KB Matosevic S. CD155 immunoregulation as a target for natural killer cell immunotherapy in glioblastoma. J Hematol Oncol. (2020) 13:76. doi: 10.1186/s13045-020-00913-2, PMID: 32532329 PMC7291472

[59] Zitti B Molfetta R Fionda C Quatrini L Stabile H Lecce M. Innate immune activating ligand SUMOylation affects tumor cell recognition by NK cells. Sci Rep. (2017) 7:10445. doi: 10.1038/s41598-017-10403-0, PMID: 28874810 PMC5585267

[60] Lv Y Sun S Zhang J Wang C Chen C Zhang Q. Loss of RBM45 inhibits breast cancer progression by reducing the SUMOylation of IRF7 to promote IFNB1 transcription. Cancer Lett. (2024) 596:216988. doi: 10.1016/j.canlet.2024.216988, PMID: 38797234

[61] Wang P Qiu J Fang Y Li S Liu K Cao Y. SENP3 inhibition suppresses hepatocellular carcinoma progression and improves the efficacy of anti-PD-1 immunotherapy. Cell Death Differ. (2025) 32:959–72. doi: 10.1038/s41418-024-01437-9IF PMC12089275 39755756

[62] Wang Z Pan B Qiu J Zhang X Ke X Shen S. SUMOylated IL-33 in the nucleus stabilizes the transcription factor IRF1 in hepatocellular carcinoma cells to promote immune escape. Sci Signal. (2023) 16:eabq3362. doi: 10.1126/scisignal.abq3362, PMID: 36917642

[63] Kumar S Schoonderwoerd MJA Kroonen JS de Graaf IJ Sluijter M Ruano D. Targeting pancreatic cancer by TAK-981: a SUMOylation inhibitor that activates the immune system and blocks cancer cell cycle progression in a preclinical model. Gut. (2022) 71:2266–83. doi: 10.1136/gutjnl-2021-324834, PMID: 35074907 PMC9554032

[64] Xiao J Sun F Wang YN Liu B Zhou P Wang FX. UBC9 deficiency enhances immunostimulatory macrophage activation and subsequent antitumor T cell response in prostate cancer. J Clin Invest. (2023) 133:e158352. doi: 10.1172/JCI158352, PMID: 36626227 PMC9927932

[65] Chen L Che Y Huang C. SENP3: cancers and diseases. Biochim Biophys Acta Rev Cancer. (2025) 1880:189260. doi: 10.1016/j.bbcan.2025.189260, PMID: 39765284

[66] Zhou J Li XY Liu YJ Feng J Wu Y Shen HM. Full-coverage regulations of autophagy by ROS: from induction to maturation. Autophagy. (2022) 18:1240–55. doi: 10.1080/15548627.2021.1984656, PMID: 34662529 PMC9225210

[67] Sun Z Hu S Luo Q Ye D Hu D Chen F. Overexpression of SENP3 in oral squamous cell carcinoma and its association with differentiation. Oncol Rep. (2013) 29:1701–6. doi: 10.3892/or.2013.2318, PMID: 23467634 PMC3658864

[68] Xiao M Bian Q Lao Y Yi J Sun X Sun X. SENP3 loss promotes M2 macrophage polarization and breast cancer progression. Mol Oncol. (2022) 16:1026–44. doi: 10.1002/1878-0261.12967, PMID: 33932085 PMC8847990

[69] Wang J Jia W Zhou X Ma Z Liu J Lan P. CBX4 suppresses CD8^+^ T cell antitumor immunity by reprogramming glycolytic metabolism. Theranostics. (2024) 14:3793–809. doi: 10.7150/thno.95748, PMID: 38994031 PMC11234269

[70] Shyu Y Liao P Huang T Yang C Lu M Huang S. Genetic disruption of KLF1 K74 SUMOylation in hematopoietic system promotes healthy longevity in mice. Adv Sci (Weinh). (2022) 9:e2201409. doi: 10.1002/advs.202201409, PMID: 35822667 PMC9443461

[71] Wu Z Huang H Han Q Hu Z Teng XL Ding R. SENP7 senses oxidative stress to sustain metabolic fitness and antitumor functions of CD8+ T cells. J Clin Invest. (2022) 132:e155224. doi: 10.1172/JCI155224, PMID: 35143421 PMC8970670

[72] Nefedova Y Cheng P Gilkes D Blaskovich M Beg AA Sebti SM. Activation of dendritic cells via inhibition of Jak2/STAT3 signaling. J Immunol. (2005) 175:4338–46. doi: 10.4049/jimmunol.175.7.4338, PMID: 16177074 PMC1351251

[73] Kortylewski M Kujawski M Wang T Wei S Zhang S Pilon-Thomas S. Inhibiting Stat3 signaling in the hematopoietic system elicits multicomponent antitumor immunity. Nat Med. (2005) 11:1314–21. doi: 10.1038/nm1325, PMID: 16288283

[74] He J Chen Y Ding H Zhou JA Xing Z Yang X. Autocrine VEGF-B signaling maintains lipid synthesis and mitochondrial fitness to support T cell immune responses. J Clin Invest. (2024) 134:e176586. doi: 10.1172/JCI176586, PMID: 39145452 PMC11324299

[75] Li JJ Xie D. RACK1, a versatile hub in cancer. Oncogene. (2015) 34:1890–8. doi: 10.1038/onc.2014.127, PMID: 24882575

[76] Wang K Xiong J Lu Y Wang L Tian T. SENP1-KLF4 signaling regulates LPS -induced macrophage M1 polarization. FEBS J. (2023) 290:209–24. doi: 10.1111/febs.16589, PMID: 35942612

[77] Hernandez-Quiles M Broekema MF Kalkhoven E. PPARgamma in metabolism, immunity, and cancer: unified and diverse mechanisms of action. Front Endocrinol (Lausanne). (2021) 12:624112. doi: 10.3389/fendo.2021.624112, PMID: 33716977 PMC7953066

[78] Negishi H Taniguchi T Yanai H. The interferon (IFN) class of cytokines and the IFN regulatory factor (IRF) transcription factor family. Cold Spring Harb Perspect Biol. (2018) 10:a028423. doi: 10.1101/cshperspect.a028423, PMID: 28963109 PMC6211389

[79] Liao X Sharma N Kapadia F Zhou G Lu Y Hong H. Krüppel-like factor 4 regulates macrophage polarization. J Clin Invest. (2011) 121:2736–49. doi: 10.1172/JCI45444, PMID: 21670502 PMC3223832

[80] Liew FY Girard JP Turnquist HR. Interleukin-33 in health and disease. Nat Rev Immunol. (2016) 16:676–89. doi: 10.1038/nri.2016.95, PMID: 27640624

[81] Hammerich-Hille S Kaipparettu BA Tsimelzon A Creighton CJ Jiang S Polo JM. SAFB1 mediates repression of immune regulators and apoptotic genes in breast cancer cells. J Biol Chem. (2010) 285:3608–16. doi: 10.1074/jbc.M109.066431, PMID: 19901029 PMC2823501

[82] Álvarez-Garcia V Tawil Y Wise HM. Leslie NR Mechanisms of PTEN loss in cancer: It’s all about diversity. Semin Cancer Biol. (2019) 59:66–79. doi: 10.1016/j.semcancer.2019.02.001, PMID: 30738865

[83] Huang C Han Y Wang Y Sun X Yan S Yeh ETH. SENP3 is responsible for HIF-1 transactivation under mild oxidative stress via p300 de-SUMOylation. EMBO J. (2009) 28:2748–62. doi: 10.1038/emboj.2009.210, PMID: 19680224 PMC2750016

[84] Hu Z Teng XL Zhang T Yu X Ding R Yi J. SENP3 senses oxidative stress to facilitate STING-dependent dendritic cell antitumor function. Mol Cell. (2021) 81:940–952.e5. doi: 10.1016/j.molcel.2020.12.024, PMID: 33434504

[85] Liu S Zhang H Li M Hu D Li C Ge B. Recruitment of Grb2 and SHIP1 by the ITT-like motif of TIGIT suppresses granule polarization and cytotoxicity of NK cells. Cell Death Differ. (2013) 20:456–64. doi: 10.1038/cdd.2012.141, PMID: 23154388 PMC3569986

[86] Llombart V Mansour MR. Therapeutic targeting of “undruggable” MYC. EBioMedicine. (2022) 75:103756. doi: 10.1016/j.ebiom.2021.103756, PMID: 34942444 PMC8713111

[87] Kukkula A Ojala VK Mendez LM Sistonen L Elenius K Sundvall M. Therapeutic potential of targeting the SUMO pathway in cancer. Cancers (Basel). (2021) 13:4402. doi: 10.3390/cancers13174402, PMID: 34503213 PMC8431684

[88] Kroonen JS Vertegaal ACO. Targeting SUMO signaling to wrestle cancer. Trends Cancer. (2021) 7:496–510. doi: 10.1016/j.trecan.2020.11.009, PMID: 33353838

[89] Canon JR Callinan A Bradley S Wang L Kui M Wein A. SB-4826, a first-in-class oral, covalent inhibitor of SUMO E1 that induces IFN signaling and inhibits tumor growth as monotherapy and in combination with immune checkpoint blockade. Cancer Res. (2023) 83:LB318. doi: 10.1158/1538-7445.AM2023-LB318

[90] Lownik J Milshteyn L Villamejor A Merchant A. Inhibition of SUMOylation reverses T-cell metabolic exhaustion in large B-cell lymphoma. Cancer Res. (2025) 85:473. doi: 10.1158/1538-7445.AM2025-473

[91] Erdem S Lee HJ Shankara Narayanan JSN Tharuka MDN de la Torre J Ren T. Inhibition of SUMOylation Induces Adaptive Antitumor Immunity against Pancreatic Cancer through Multiple Effects on the Tumor Microenvironment. Mol Cancer Ther. (2024) 23:1597–612. doi: 10.1158/1535-7163.MCT-23-0572, PMID: 39150446 PMC11534524

[92] Wang Z Pan B Su L Yu H Wu X Yao Y. SUMOylation inhibitors activate anti-tumor immunity by reshaping the immune microenvironment in a preclinical model of hepatocellular carcinoma. Cell Oncol (Dordr). (2024) 47:513–32. doi: 10.1007/s13402-023-00880-z, PMID: 38055116 PMC12974044

[93] Nakamura A Grossman S Song K Xega K Zhang Y Cvet D. The SUMOylation inhibitor subasumstat potentiates rituximab activity by IFN1-dependent macrophage and NK cell stimulation. Blood. (2022) 139:2770–81. doi: 10.1182/blood.2021014267, PMID: 35226739 PMC11022956

[94] Lu Z McBrearty N Chen J Tomar VS Zhang H De Rosa G. ATF3 and CH25H regulate effector trogocytosis and anti-tumor activities of endogenous and immunotherapeutic cytotoxic T lymphocytes. Cell Metab. (2022) 34:1342–1358.e7. doi: 10.1016/j.cmet.2022.08.007, PMID: 36070682 PMC10496461

[95] Kroonen JS Wouters AK de Graaf IJ Remst DFG Kumar S Wachsmann TLA. Targeting epigenetic regulation and post-translational modification with 5-Aza-2’ deoxycytidine and SUMO E1 inhibition augments T-cell receptor therapy. J Immunother Cancer. (2024) 12:e008654. doi: 10.1136/jitc-2023-008654, PMID: 39326886 PMC11425949

[96] Derry JMJ Burns C Frazier JP Beirne E Grenley M DuFort CC. Trackable intratumor microdosing and spatial profiling provide early insights into activity of investigational agents in the intact tumor microenvironment. Clin Cancer Res. (2023) 29:3813–25. doi: 10.1158/1078-0432.CCR-23-0827, PMID: 37389981 PMC10502463

[97] Dudek A Juric D Dowlati A Vaishampayan U Assad H Rodón J. First-in-human phase 1/2 study of the first-in-class sumo-activating enzyme inhibitor tak-981 in patients with advanced or metastatic solid tumors or relapsed/refractory lymphoma: phase 1 results. J Immunother Cancer. (2021) 9:A505–6. doi: 10.1136/jitc-2021-SITC2021.476

[98] Saggu G Stroopinsky D Dudek AZ Olszanski AJ Juric D Dowlati A. Subasumstat, a first-in-class inhibitor of SUMO-activating enzyme, demonstrates dose-dependent target engagement and SUMOylation inhibition, leading to rapid activation of innate and adaptive immune responses in the dose escalation portion of a phase 1/2 clinical study. Eur J Cancer. (2022) 174:S125–6. doi: 10.1016/S0959-8049(22)01134-0

